# Supplementary material for: XCP1 cleaves Pathogenesis-related protein 1 into CAPE9 for systemic immunity in Arabidopsis
Source: Nat Commun. 2023 Aug 4;14:4697. doi: 10.1038/s41467-023-40406-7 (PMC10403534; doi:10.1038/s41467-023-40406-7)
Supplement: Supplementary file 1 — Supplementary Information [file 41467_2023_40406_MOESM1_ESM.pdf]

Supplementary Information

Supplementary Tables and Figures

Supplementary Table 1. Putative untruncated and truncated form sizes of *Arabidopsis* papain-like cysteine proteases.

AT Gene #	Protein Name	PLCP Subfamily[a]	Putative Protein Size[b] (kDa) (Untruncated / truncated form)
At4g36880	RDL1	I	39.40 / 25.20
At3g19400	RDL2	I	37.55 / 25.16
At3g43960	RDL3	I	38.76 / 27.10
At4g11310	RDL4 (CP1)	I	37.67 / **24.68**
At4g11320	RDL5 (CP2)	I	38.35 / **24.64**
At4g23520	RDL6	I	**36.81** / **24.41**
At1g47128	RD21A	I	**35.40** / **23.30**
At5g43060	RD21B	I	**35.33** / **23.26**
At4g19390	RD21C	I	**34.41** / **23.25**
At5g50260	CEP1	II	38.43 / 25.73
At3g48340	CEP2	II	38.12 / 25.19
At3g48350	CEP3	II	38.71 / 26.03
**At4g35350**	**XCP1**	III	**36.80** / **23.79**
At1g20850	XCP2	III	37.08 / **23.77**
At1g09850	XBCP3	IV	39.00 / **23.43**
At1g06260	THI1	V	**34.54** / **23.22**
At5g45890	SAG12	VI	**35.37** / -
At2g34080	PAP1	VI	**35.76** / -
At1g29090	PAP2	VI	**36.36** / -
At1g29080	PAP3	VI	**36.11** / -
At2g27420	PAP4	VI	**36.50** / -
At3g49340	PAP5	VI	**35.65** / -
At4g39090	RD19A	VII	37.85 / 25.12
At2g21430	RD19B	VII	37.12 / **24.70**
At4g16190	RD19C	VII	39.14 / 25.35
At3g54940	RD19D	VII	37.81 / 25.07
At5g60360	AALP	VIII	**36.95** / **23.42**
At3g45310	ALP2	VIII	37.41 / **23.65**
At1g02300	CTB1	IX	37.27 / 29.21
At1g02305	CTB2	IX	**34.75** / 26.64
At4g01610	CTB3	IX	**34.94** / 26.53

[a] There are 9 subfamilies of *Arabidopsis* papain-like cysteine proteases (PLCPs) classified by their domian structures. Here was indicated as I to IX. (*Plant Physiol.* 2012, Vol. 158, pp. 1583–1599)

[b] The putative protein sizes of PLCPs are represented for their untruncated and truncated forms reported in UniProt (https://www.uniprot.org/). Red color indicates the selected protease size ranging from 34 kDa to 37 kDa for untruncated form and 23 kDa to 25 kDa for truncated form.

Supplementary Table 2. List of *Arabidopsis* T-DNA insertion mutants

Name	Gene	Mutant Line
xcp1	At4g35350	SALK_084789
xcp2	At1g20850	SALK_010938
rd19b	At2g21430	SALK_095569
sag12	At5g45890	SALK_095721
aalp	At5g60360	SALK_075550
rd21c	At3g19390	SALK_058300
thi1	At1g06260	SALK_056849
rd19a	At4g39090	SALK_031088
ctb3	At4g01610	SALK_019630

Supplementary Table 3. Primers used in this study

**Primers for cloning**	
PR1_F'	ATG AAT TTT ACT GGC TAT TCT CGA TTT TT
PR1-D150A/P151A_F'	ATG AAT TTT ACT GGC TAT TCT CGA TTT TTA ATC GTC TTT GTA GCT CTT GTA GG
PR1-D150A_R'	GTA TGG CTT CTC GTT CAC ATA ATT CCC ACG AGG AGC ATA GTT GCA ACT G
PR1-P151A_R'	GTA TGG CTT CTC GTT CAC ATA ATT CCC ACG CGC ATC ATA GTT GC
PR1_CDS_R’	TTA GTA TGG CTT CTC GTT CAC
CAPE9-truncated PR1_R’	TTA ATC ATA GTT GCA ACT GAT TAT
PR1-BsF1 (for CRISPR/Cas9)	ATA TAT GGT CTC GAT TGT CCC ATG CAG TGG GAC GAG AGT T
PR1-F10 (for CRISPR/Cas9)	TGT CCC ATG CAG TGG GAC GAG AGT TTT AGA GCT AGA AAT AGC
DT0-BsR2 (for CRISPR/Cas9)	ATA TTA TTG GTC TCA ATC TCT TAG TCG ACT CTA CCA AT
PR1-BsF2 (for CRISPR/Cas9)	ATA TTA TTG GTC TCA AGA TTG TAG CCC ACA AGA TTA TCT AAG TT
PR1-F20 (for CRISPR/Cas9)	TGT AGC CCA CAA GAT TAT CTA AGT TTT AGA GCT AGA AAT AGC
PR1-R0 (for CRISPR/Cas9)	AAC ACC AGA GTG TAT GAG TCT GCC AAT CAC TAC TTC GTC TCT AAC CAT
PR1-BsR3 (for CRISPR/Cas9)	ATT ATT GGT CTC GAA ACA CCA GAG TGT ATG AGT CTG CC
XCP1-His_F'	ATG GCT TTT TCT GCA CCA TCA CTT TCC
XCP1-His_R1'	GAT GAT GAT GCT TGG TCT TGG TAG GAT AT
XCP1-His_R2'	TCA ATG ATG ATG ATG ATG ATG CTT GGT CT
XCP1_promoter_F'	AAA AGC CTG CAG GGT GTT TGC ACT TTG CA
XCP1_promoter_R'	AGC CAT GGG CCC CCA AAT TTG TTC ACT
XCP1-C161A_F'	GGT CAA TGT GGT AGC GCT TGG GCA TTT TCA ACA
XCP1-C161A_R'	CTG TTG AAA ATG CCC AAG CGC TAC CAC ATT GAC
**Primers for gene expression level**	
PR1_RT-PCR_F'	GTG CTC TTG TTC TTC CCT CG
PR1_RT-PCR_R'	GCC TGG TTG TGA ACC CTT AG
XCP1_RT-PCR_F'	GAT ACA CGC CGG AGC ATT TG
XCP1_RT-PCR_R'	GTA GCC GCT GAT TGT CAC AC
XCP1_qPCR_F'	GCT CAT CAG CCA GTC AGT G
XCP1_qPCR_R'	ACC ATT AAA CAC TCC CCC TTT GT
PR1-eYFP_RT-PCR_R’	GAA CTT CAG GGT CAG CTT GCC GTA
ACTIN2_RT/qPCR_F '	GGT AAC ATT GTG CTC AGT GGT GG
ACTIN2_RT/qPCR_R'	GGT GCA ACG ACC TTA ATC TTC AT
**Primers for genotyping**	
LbB. 1.3	ATT TTG CCG ATT TCG GAA C
PR1-F	ATG AAT TTT ACT GGC TAT TCT CGA TTT TT
PR1-R	GTA TGG CTT CTC GTT CAC ATA ATT C
8484-F	TTT TCC CAG TCA CGA CGT TGT
8485-R	GTC CTT GTA TAA ACG CAA TG
SV40 NLS-F	CCG AAG AAG AAG AGG AAG GTT
Cas9-middle-R	CGT CTT CCC GGA CTG CTT
XCP1_LP	GAA GCC TCA ATA GCC ACA CTG
XCP1_RP	TAT GGC TTT TTC TGC ACC ATC
XCP2_LP	AAA GGG AAA AGC TAC TGG CTC
XCP2_RP	GGT TTC CCA GTG TTC CTC TTC
CTB3_LP	TTG TGT GTG TGT GTT GAC TGC
CTB3_RP	AAA ACT TAC ATC ACC CCA GCC
AALP_LP	TAG TTT TGG TGG TTC TCG TCG
AALP_RP	CTG ATG GTA AGC TGC CTC AAG
RD19A_LP	AAC CTC GTG ATC ACA GTC GAC
RD19A_RP	AGA CAA CAC GGC ACT GCT AAC
mRD19B_LP	CTG ACT CGT TCT GAG TTT CGG
mRD19B_RP	TGG CAT TGT TGT TTC GAA TTC
mRD21C_LP	GTA CCT TCC GGG AAT GGT TAG
mRD21C_RP	CAT AAG ACC TCC ACC ACA TCC
SAG12_LP	ACT GTT CAA TGG AGC GAA TTG
SAG12_RP	CAA AAA GAC CAA TCC AAA AGC
THI1_LP	CAA AGA GAT GGT GAG CAA AGC
THI1_RP	CAA GCT ATC CCT TGC AGT GAG

Mascot Search Results

Peptide View

MS/MS Fragmentation of **PRGNYVNEKPY**
Found in **AT2G14610.1**, | pathogenesis-related protein 1 | Chr2:6241944-6242429 REVERSE LENGTH=161 | 201606

Match to Query 6336: 1335.657632 from(668.836092,2+) intensity(1713972.2500)
Title: 200220_Cindee_QEHF_Arab_Pep_SA_DDA2_F16.6503.6503.2 File:"200220_Cindee_QEHF_Arab_Pep_SA_DDA2_F16.raw", NativeID:"controllerType=0 controllerNumber=1 scan=6503"
Data file D:\files\experiments\datas\MSdata\QEHF\2019-2021\210913_reanalyze cindee data SA24h arab pep\mgf (nonuniqua)\200220_Cindee_QEHF_Arab_Pep_SA_DDA2_F16.mgf

Click mouse within plot area to zoom in by factor of two about that point
Or, Plot from 50 to 1100 Da Full range
Label all possible matches ○ Label matches used for scoring ◉

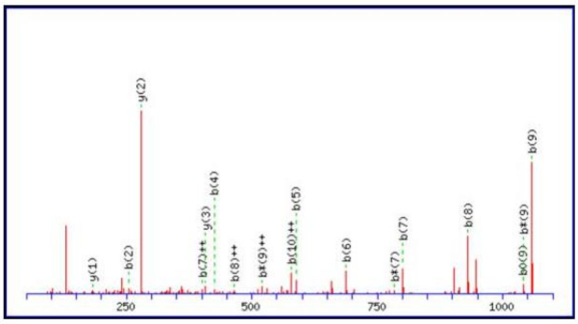


```
Monoisotopic mass of neutral peptide Mr(calc): 1335.657120
Ions Score: 33  Expect: 0.017
Matches : 17/94 fragment ions using 40 most intense peaks   (help)
```

#	b	b^{++}	b*	b^{*++}	b^0	b^{0++}	Seq.	y	y^{++}	y*	y^{*++}	y^0	y^{0++}	#
1	98.060040	49.533658					P							11
2	*254.161151*	127.584213	237.134602	119.070939			R	1239.611662	620.309469	1222.585113	611.796195	1221.601097	611.304187	10
3	311.182615	156.094945	294.156066	147.581671			G	1083.510551	542.258914	1066.484002	533.745639	1065.499986	533.253631	9
4	*425.225542*	213.116409	408.198993	204.603135			N	1026.489087	513.748182	1009.462538	505.234907	1008.478522	504.742899	8
5	*588.288871*	294.648074	571.262322	286.134799			Y	912.446160	456.726718	895.419611	448.213443	894.435595	447.721435	7
6	*687.357285*	344.182281	670.330736	335.669006			V	749.382831	375.195054	732.356282	366.681779	731.372266	366.189771	6
7	*801.400212*	**401.203744**	784.373663	392.690470			N	650.314417	325.660847	633.287868	317.147572	632.303852	316.655564	5
8	*930.442805*	**465.725041**	913.416256	457.211766	912.432240	456.719758	E	536.271490	268.639383	519.244941	260.126109	518.260925	259.634101	4
9	*1058.537768*	529.772522	1041.511219	521.259248	1040.527203	520.767239	K	**407.228897**	204.118086	390.202348	195.604812			3
10	1155.590532	**578.298904**	1138.563983	569.785630	1137.579967	569.293621	P	**279.133934**	140.070605					2
11							Y	**182.081170**	91.544223					1

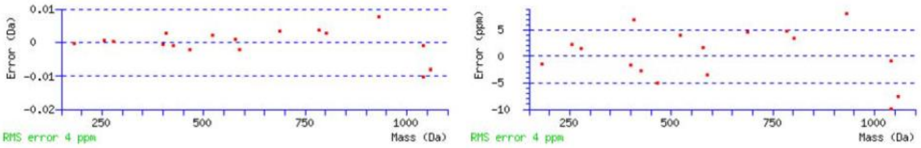

NCBI **BLAST** search of PRGNYVNEKPY
(Parameters: blastp, nr protein database, expect=20000, no filter, PAM30)
Other BLAST web gateways

All matches to this query

Score	Mr(calc):	Delta	Sequence
33.4	1335.657120	0.000512	PRGNYVNEKPY
13.7	1335.652664	0.004968	QDKGKMPMSVTV
13.5	1334.654678	1.002954	KENCLGKERCR
13.5	1335.656647	0.000985	KKMESFYKLM
12.4	1335.656662	0.000970	KGIWCVMIELE
11.7	1335.660492	-0.002860	AFKSPGADRKME
9.6	1334.649994	1.007638	TSSISNPNKMKP
7.9	1334.649979	1.007653	EEITQSIEKRC
7.3	1335.655777	0.001855	KSEPETEEFIK
7.3	1335.663010	-0.005378	QLRSQTSESLTS

Supplementary Fig. 1. Identification result of endogenous AtCAPE9 using Mascot MS/MS ion matching.
The delta score (DS) for the endogenous AtCAPE9 was 19.7 (1st ranked matching score - 2nd ranked matching score, 33.4 - 13.7 = 19.7)

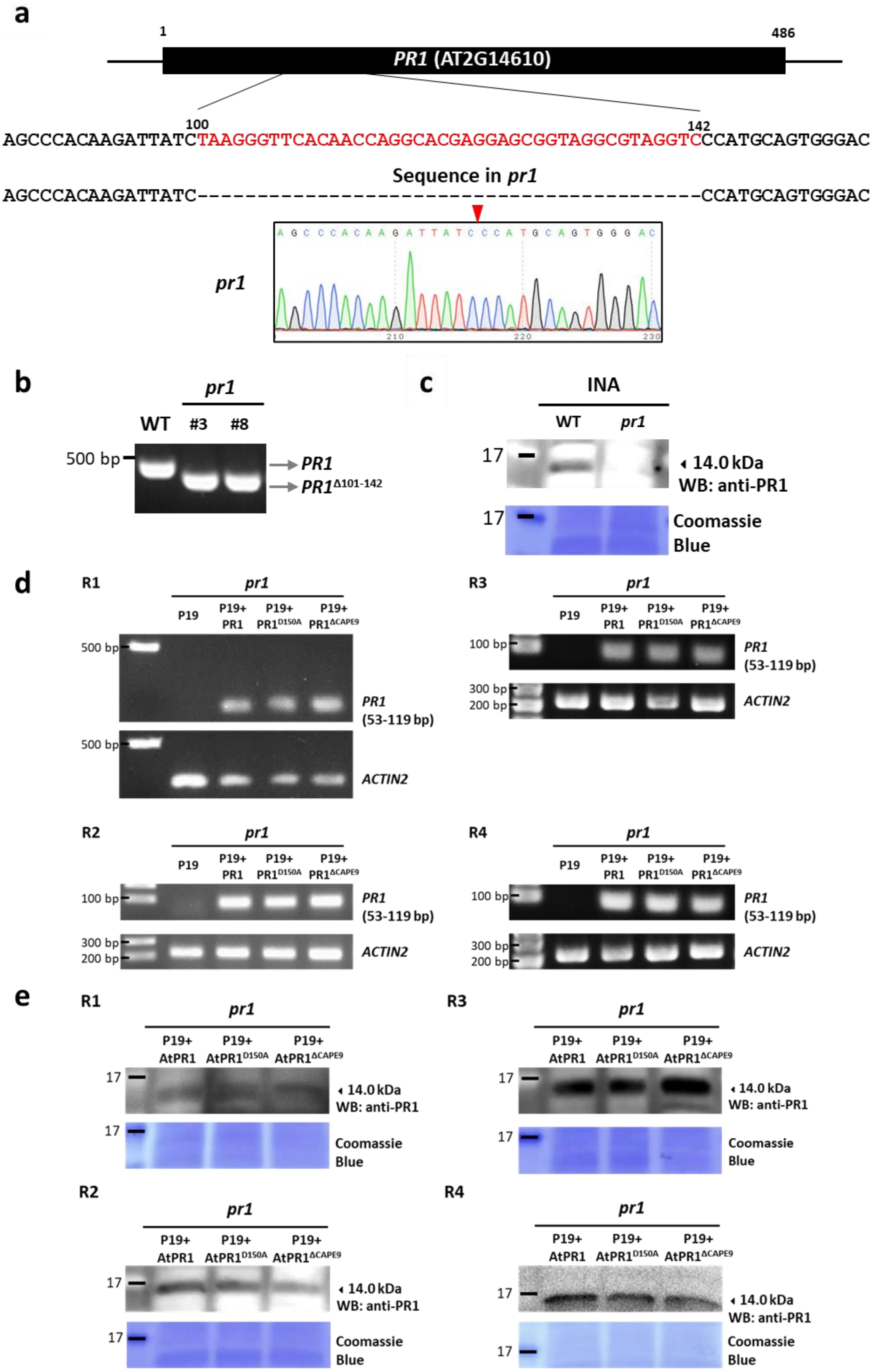

Supplementary Fig. 2. Examination of *PR1* sequence and expression in *pr1* mutant with or without local transformation of native or mutated *PR1* by *Agrobacterium*.
(**a**) Sequence of the truncated region (101-142 bp) of *PR1* (At2g14610) in the *pr1 Arabidopsis* mutant

generated by CRISPR/Cas9. Experiments were repeated for three times with similar results. (**b**) Genotyping of *PR1* in WT and T2 lines (#3 and #8) of *pr1* mutant. (**c**) Immunoblot of PR1 in WT and *pr1* mutant using anti-PR1. The plants were treated by 60 μM INA for 60 min. (**b**, **c**) Experiments were repeated for two times with similar results. (**d**) RT-PCR analysis of *PR1* using the primers targeting 53-119 bp of *PR1*. *ACTIN2* was used as internal control. (**e**) Immunoblot of PR1 in *pr1* infiltrated with *Agrobacterium* carrying *P19* plasmid together with *Agrobacterium* carrying native *PR1* (P19+PR1), alanine-substituted *PR1* (P19+PR1^{D150A}), or CAPE9-truncated *PR1* (P19+PR1$^{\Delta CAPE9}$) driven by 35S promotor. The sizes of native and mutated PR1 proteins were estimated to be 13~14 kDa, detected by western blotting with anti-PR1 antibody. Coomassie Blue staining shows total protein loaded. (**d**, **e**) Experiments were repeated for four times with similar results.

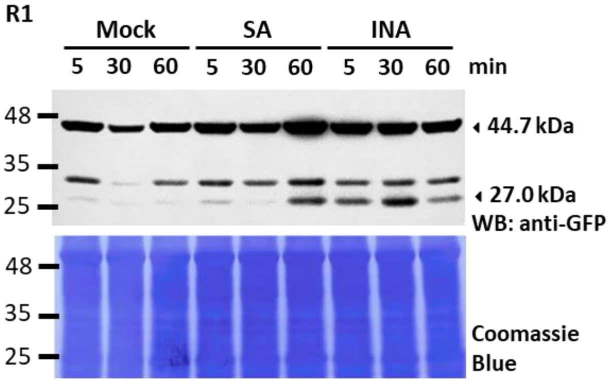

Replicate 1 (R1)	Mock (Control)	SA	INA
Time (min)	60	60	60
Band intensity of PR1-eYFP	15734	31390	17983
Band intensity of CAPE9-eYFP	335	8156	4058
%Cleavage [CAPE9-eYFP/ (PR1-eYFP+CAPE9-eYFP)]	2.1	20.6	18.4
Fold change (Experimental vs. Control)	1.0	9.9	8.8
Log_2 Fold change (Experimental vs. Control)	0.0	3.3	3.1

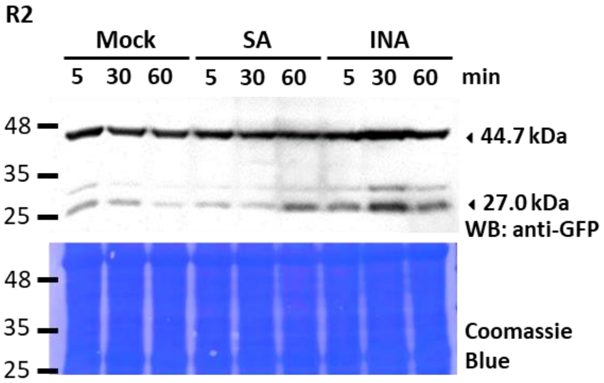

Replicate 2 (R2)	Mock (Control)	SA	INA
Time (min)	60	60	60
Band intensity of PR1-eYFP	11056	15875	13347
Band intensity of CAPE9-eYFP	997	7587	7817
%Cleavage [CAPE9-eYFP/ (PR1-eYFP+CAPE9-eYFP)]	8.3	32.3	36.9
Fold change (Experimental vs. Control)	1.0	3.9	4.5
Log_2 Fold change (Experimental vs. Control)	0.0	2.0	2.2

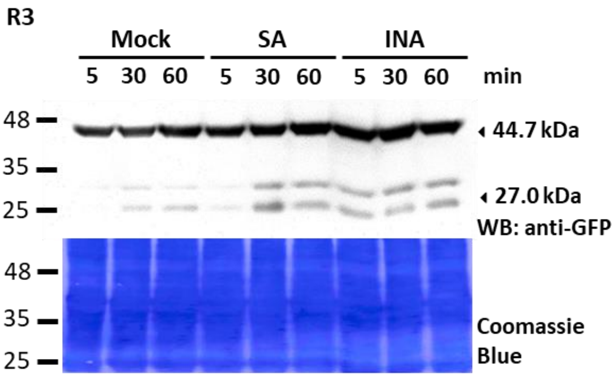

Replicate 3 (R3)	Mock (Control)	SA	INA
Time (min)	60	60	60
Band intensity of PR1-eYFP	16382	19789	18130
Band intensity of CAPE9-eYFP	361	2808	3063
%Cleavage [CAPE9-eYFP/ (PR1-eYFP+CAPE9-eYFP)]	2.2	12.4	14.5
Fold change (Experimental vs. Control)	1.0	5.8	6.7
Log_2 Fold change (Experimental vs. Control)	0.0	2.5	2.7

Supplementary Fig. 3. Immunoblot of PR1-eYFP and AtCAPE9-eYFP fragment in transgenic *PR1-eYFP Arabidopsis* seedlings with Mock, SA or INA treatment.

The seedlings were immersed in medium with 10 mM $MgSO_4$ (Mock), 60 μM INA in 10 mM $MgSO_4$ (INA), or 60 μM SA in 10 mM $MgSO_4$ (SA) for 5, 30 and 60 min. The sizes of the intact/uncleaved PR1-eYFP and the putative AtCAPE9-eYFP fragment were estimated to be ~44.7 and ~27.0 kDa, respectively. The percentage of the PR1-eYFP cleavage to produce AtCAPE9-eYFP fragment (noted as %Cleavage) was calculated by the band intensity of AtCAPE9-eYFP divided by the sum of the PR1-eYFP and AtCAPE9-eYFP band intensities [(AtCAPE9-eYFP)/(PR1-eYFP +AtCAPE9-eYFP)], detected by immunoblot using anti-GFP. Coomassie Blue staining shows total protein loaded. The band intensities in immunoblots were measured by ImageJ.

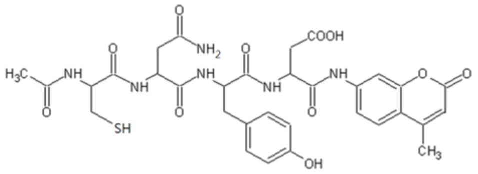

Ac-CNYD-AMC

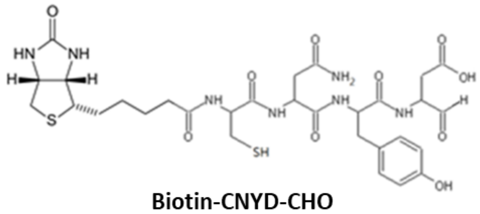

Biotin-CNYD-CHO

Supplementary Fig. 4. Design of fluorogenic protease substrate and affinity aldehyde inhibitor based on the CNYD domain.

Structure of fluorogenic protease substrate and affinity aldehyde inhibitor, shown as Ac-CNYD-AMC and biotin-CNYD-CHO, respectively.

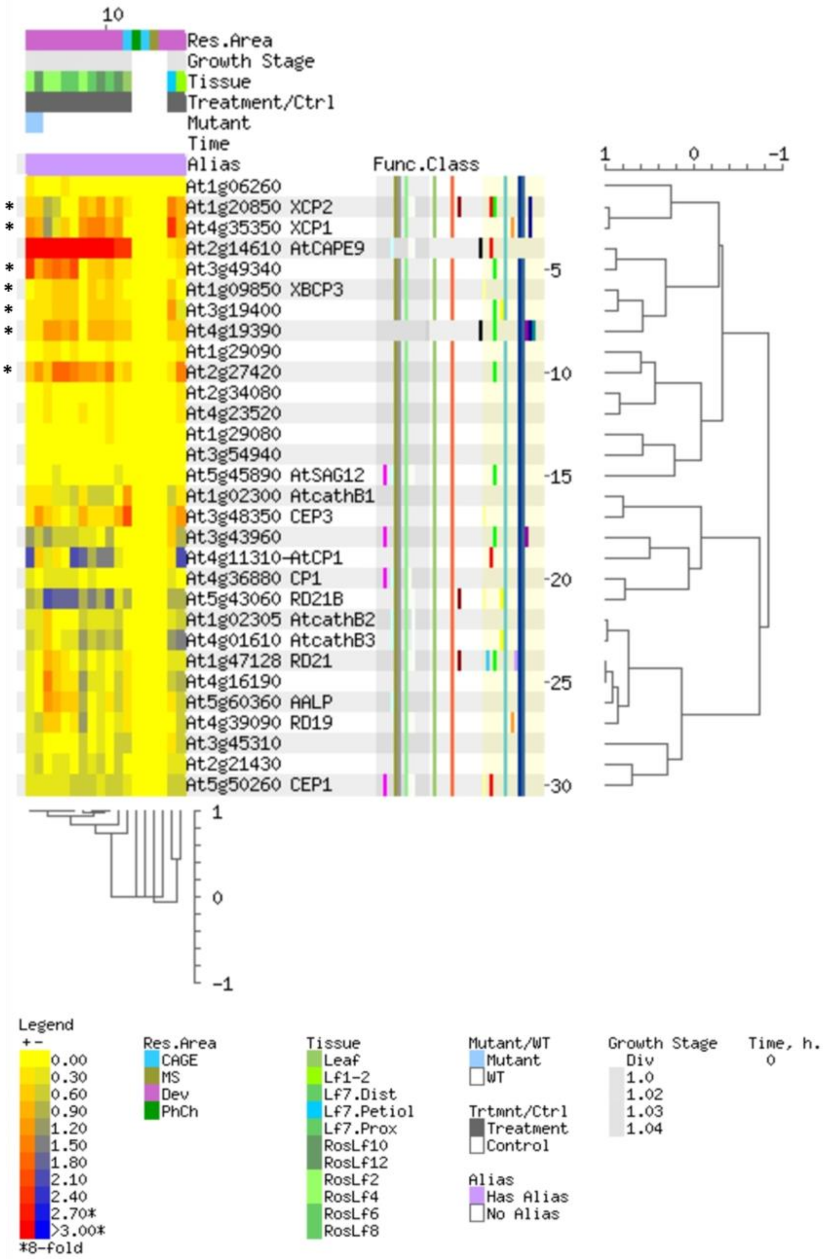

Supplementary Fig. 5. Relative expression of papain-like cysteine proteases and PR1 (AtCAPE9) at different leaf developmental stages.

The color scale indicates the log_2 of gene expression level above or below the median. Yellow shows the median value. Red indicates the gene is strongly expressed relative to the median expression levels, while dark blue indicates those poorly expressed. The expression pattern of *PR1* (labeled AtCAPE9/At2g14610) was hierarchically clustered with PLCP expression at all leaf stages to uncover potential correlations and, in turn, ESCAPE candidates (labeled with an asterisk). Data obtained from NASCArrays and the AtGenExpress Consortium (At3g48340 is not available) and analyzed by the Expression browser (*Plant J.* 2012, Vol. 43, pp. 153–163).

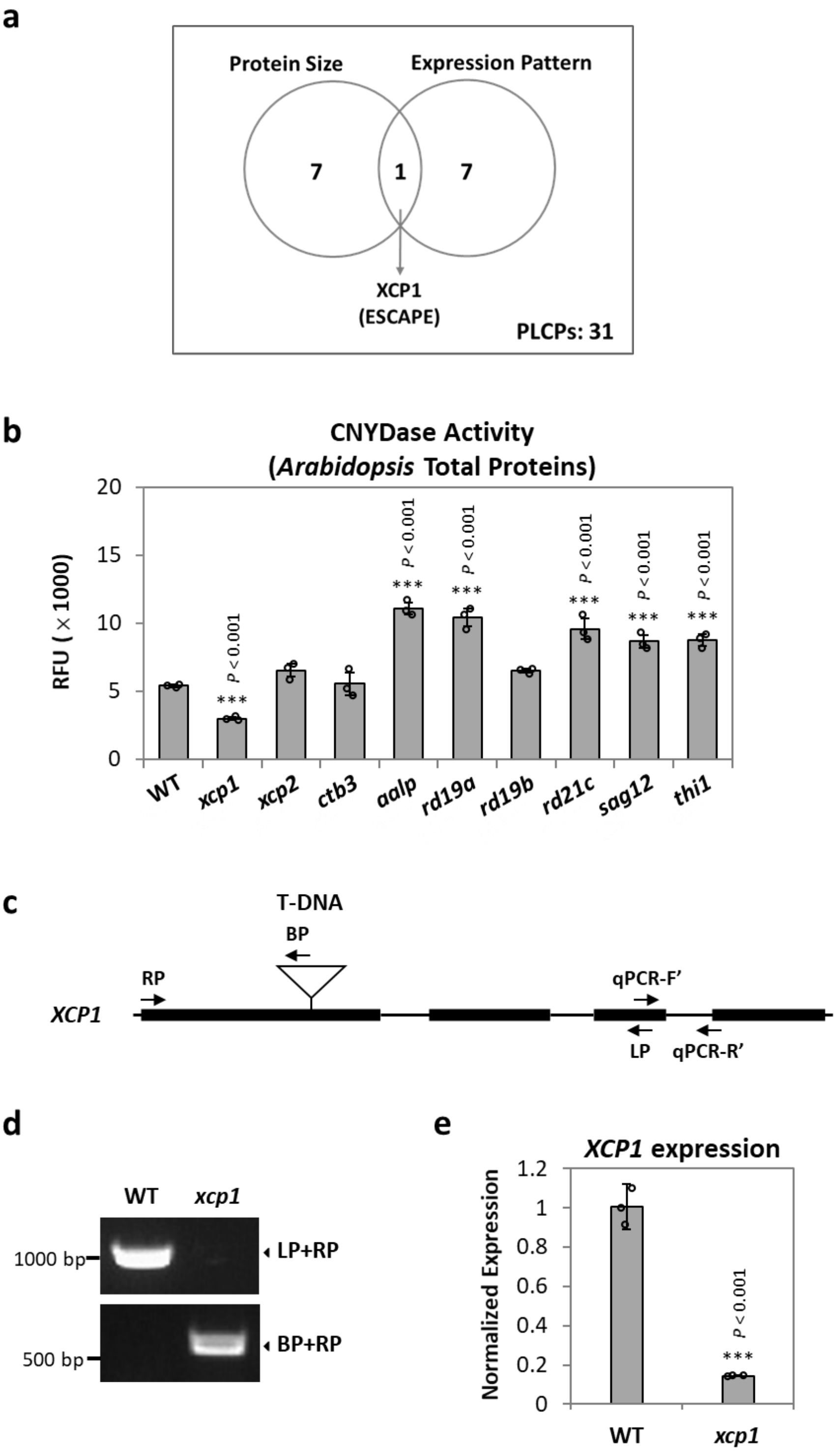

Supplementary Fig. 6. Selection of ESCAPE from PLCPs and validation for the selected ESCAPE mutant.

(**a**) Venn diagram of the ESCAPE candidates within the group of PLCPs, selected by their protein sizes (Supplementary Table 1) and expression patterns (Supplementary Fig. 5). (**b**) CNYDase activity of the selected papain-like cysteine protease mutants of *Arabidopsis*. The proteolytic activity assay was performed by incubating 50 µg protein extracted from three seedlings with 25 µM Ac-CNYD-AMC substrate. RFU of the cleaved fluorophore was measured after 5 h incubation with the substrate. Values are means ± SD of three biological replicates. Each replicate was obtained from three seedlings. *P*

values were calculated by one-tailed unpaired *t*-test (**, $P < 0.01$). The stock number of these T-DNA insertion mutants were listed in Supplementary Table 2. (**c**) A scheme exhibited for T-DNA insertion site of *XCP1* gene in the T-DNA insertion *Arabidopsis* mutant (*xcp1*; SALK_084789) and the primers designed for genotyping and quantifying the gene expression of *XCP1* in *xcp1* mutant. (**d**) The *xcp1* T-DNA insertion mutant was confirmed by genotyping. (**e**) The expression level of the *XCP1* gene in WT and *xcp1* determined by RT-qPCR. The expression of *ACTIN2* was used to normalize *XCP1* gene expression. Values are means ± SD of three biological replicates. *P* values were calculated by one-tailed unpaired *t*-test (***, $P < 0.001$).

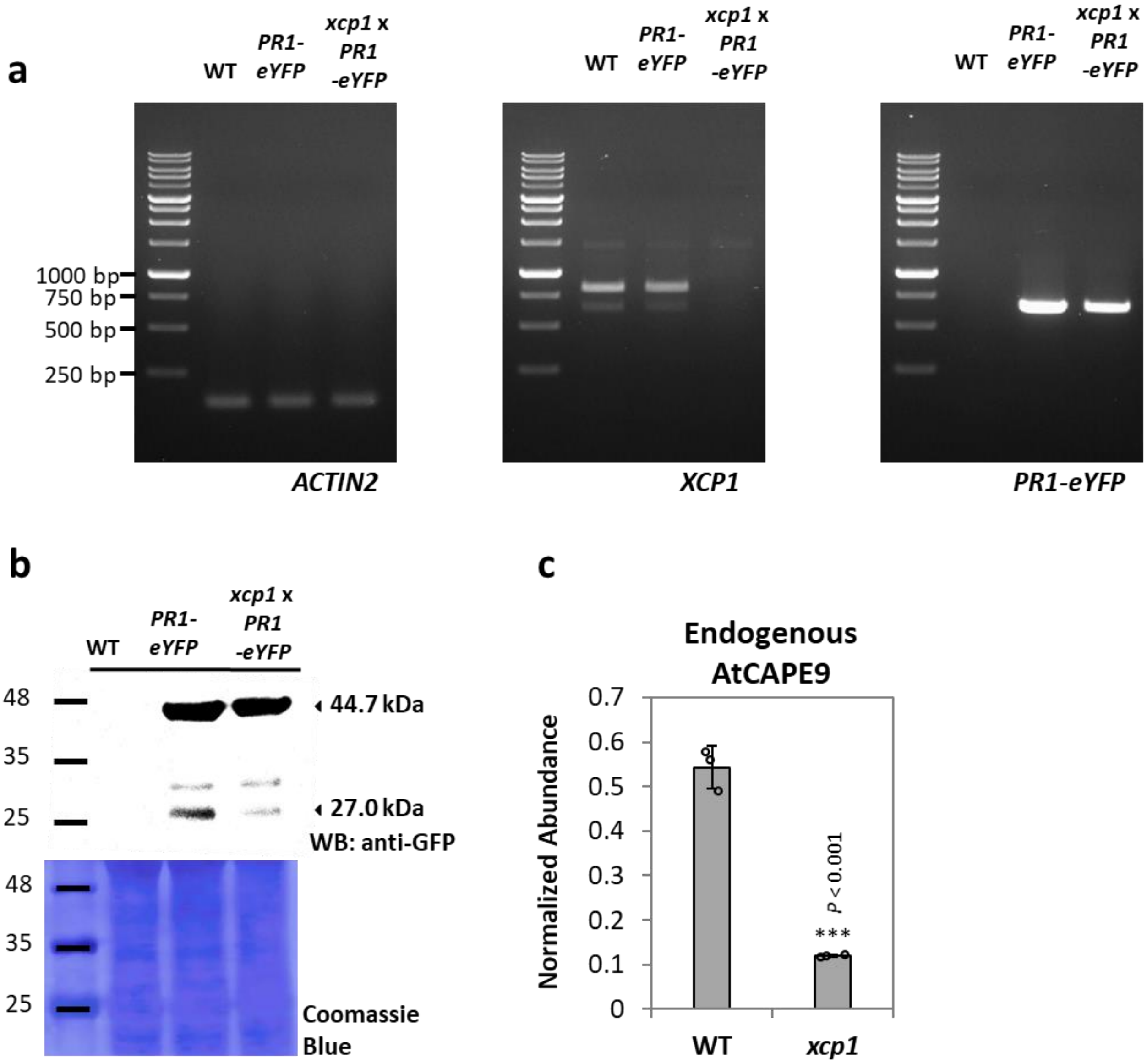

Supplementary Fig. 7. Examination of the PR1 cleavage in *xcp1* × *PR1-eYFP* line and the AtCAPE9 abundance in *xcp1* mutant.

(**a**) RT-PCR analysis was performed for detection of *ACTIN2*, *XCP1* and *PR1-eYFP* gene expression between WT, *PR1-eYFP* and the crossed line of *xcp1* × *PR1-eYFP* plants. Experiments were repeated for two times with similar results. (**b**) Immunoblot of the protein extract from WT, *PR1-eYFP* and the crossed line of *xcp1* × *PR1-eYFP* plants. The sizes of the intact PR1-eYFP and the putative AtCAPE9 fragment were estimated to be ~44.7 and ~27.0 kDa, respectively, detected by the anti-GFP. Coomassie Blue staining shows total protein loaded. Experiments were repeated for three times with similar results. (**c**) The abundance of endogenous AtCAPE9 in the *Arabidopsis* leaves with SA treatment between WT or *xcp1* plants. The abundance of AtCAPE9 was quantified and normalized by the abundance of a spiked internal control angiotensin I using LC-MS/MS analysis. Values are means ± SD of three technical replicates obtained from the analysis of a peptide mixture extracted from 5 g leaf tissues derived from a pool of five plants. *P* values were calculated by one-tailed unpaired *t*-test (***, $P < 0.001$).

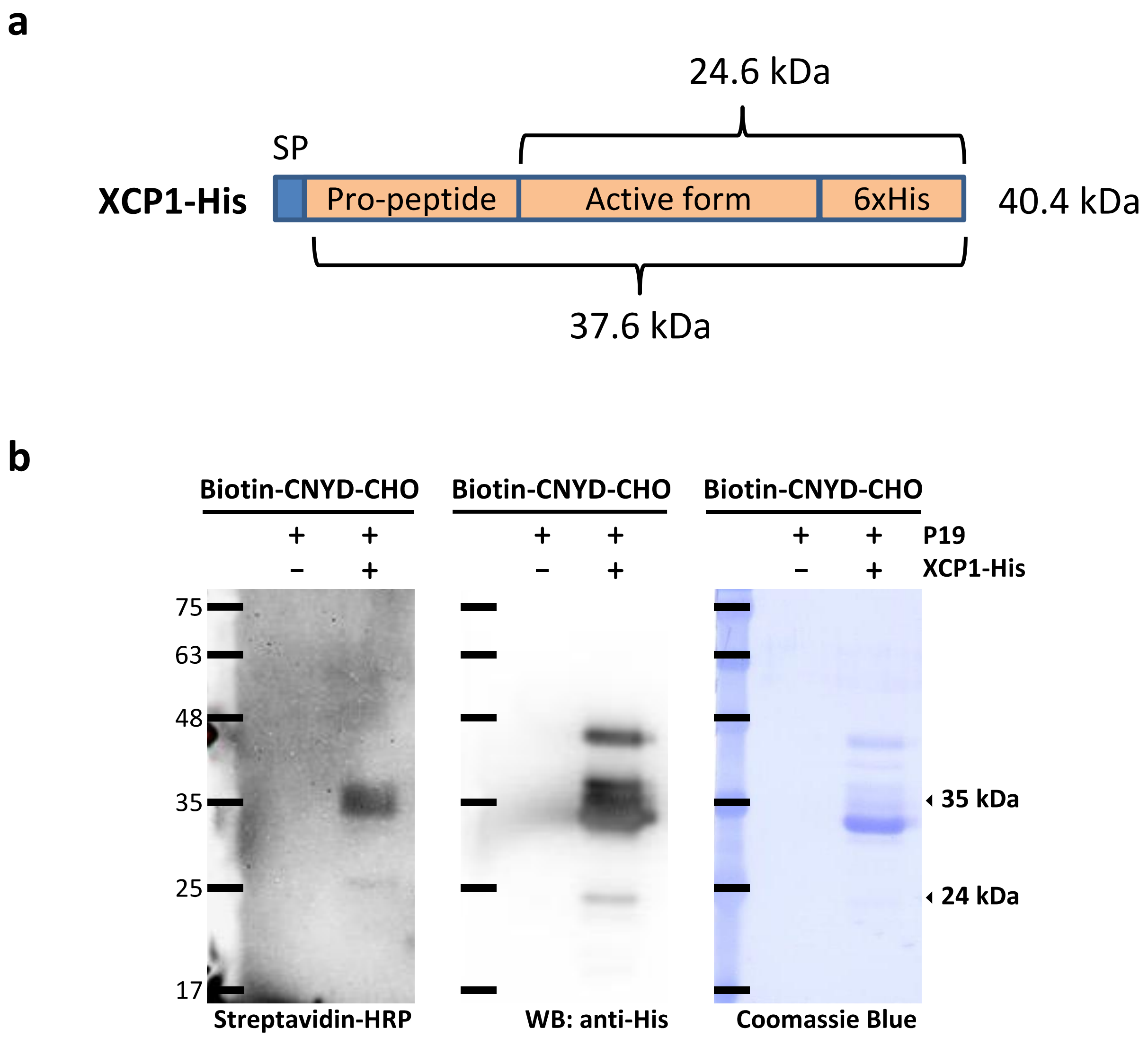

Supplementary Fig. 8. Characterization of *Arabidopsis* XCP1-His purified from the *XCP1-His*-overexpressed *Nicotiana benthamiana*.

(**a**) A scheme of *Arabidopsis* XCP1-His protein showing the molecular weight of the untruncated and truncated form. (**b**) Analysis of Ni-NTA purified proteins from the *Nicotiana benthamiana* leaves overexpressing *P19* or *P19* with *XCP1-His* genes. Purified proteins were labeled with biotin-CNYD-CHO and analyzed by immunoblot using anti-His antibody or streptavidin-HRP. Coomassie Blue staining shows all purified protein loaded. Experiments were repeated for three times with similar results.

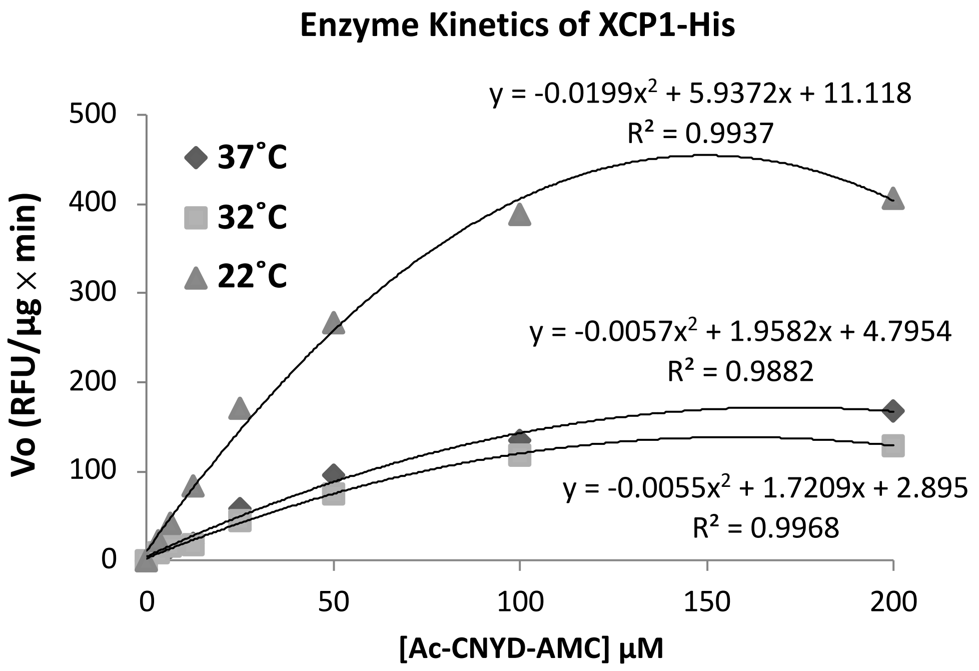

Temp.	22°C	32°C	37°C
**Vmax** (RFU/(µg × min))	407.01	129.24	168.86
**Km = [S]** (µM)	36.99	41.33	47.14

Supplementary Fig. 9. Determination of the enzyme kinetics of *Arabidopsis* XCP1-His based on CNYDase activity under different temperatures.

The V_{max} and K_m of proteolytic activity were determined by 2.5 h incubation of 1 µg purified XCP1-His with different concentrations of CNYD substrate at 22, 32 or 37°C.

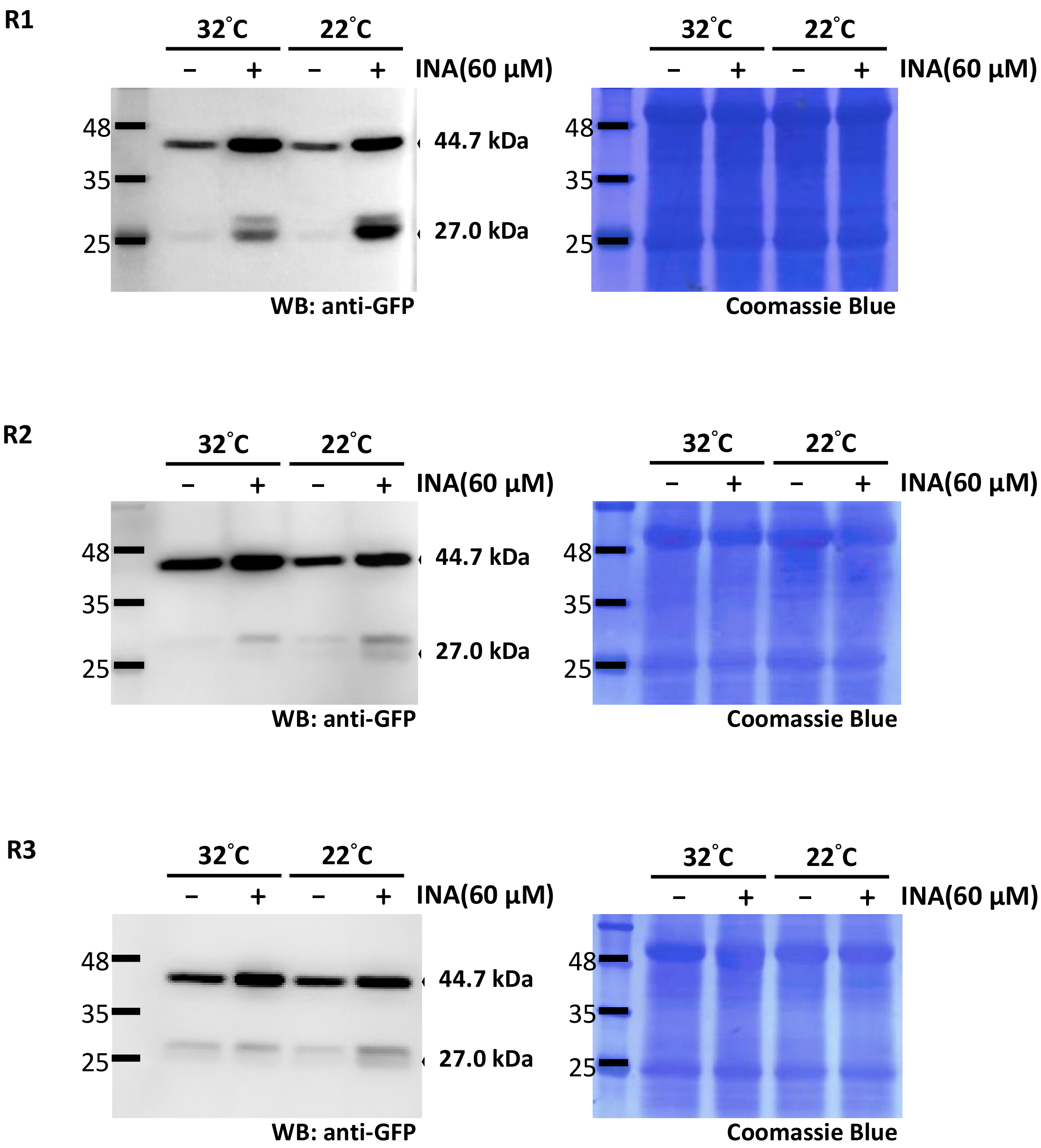

Supplementary Fig. 10. Immunoblot of PR1-eYFP and AtCAPE9-eYFP fragment in transgenic *PR1-eYFP Arabidopsis* seedlings with Mock or INA treatment at 32 or 22°C.
The seedlings were pre-incubated at 32 or 22°C before treatments for 30 min and then immersed in medium with 10 mM $MgSO_4$ (Mock) or 60 µM INA in 10 mM $MgSO_4$ (INA) for 60 min. The sizes of the intact/uncleaved PR1-eYFP and the putative AtCAPE9-eYFP fragment were estimated to be ~44.7 and ~27.0 kDa, respectively, detected by immunoblot using anti-GFP. Coomassie Blue staining shows total protein loaded.

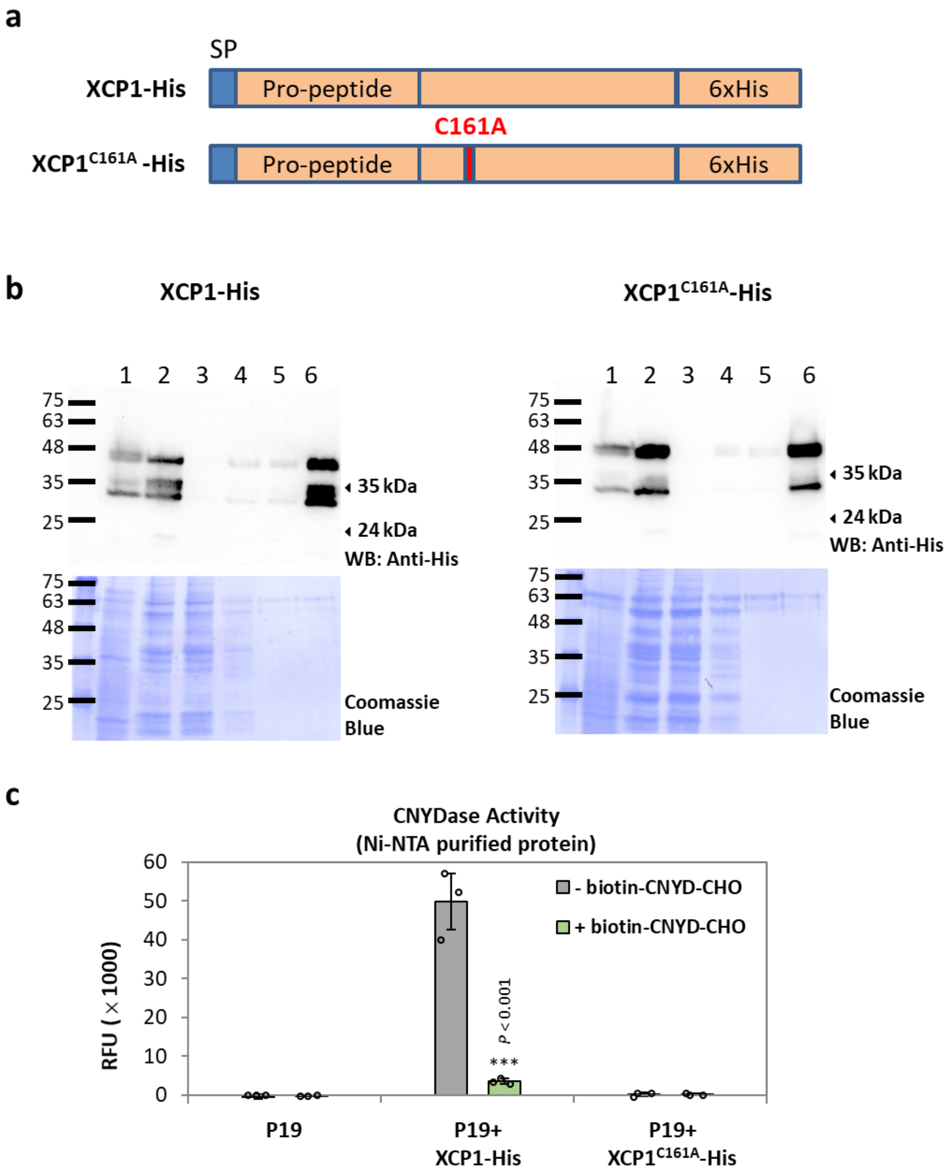

Supplementary Fig. 11. Examination of the alanine-substituted mutation on the putative active site of *Arabidopsis* XCP1-His for CNYDase activity.
(**a**) The schemes represent the original and alanine-substituted (C161A) XCP1-His protein sequences. (**b**) Analysis of Ni-NTA purified proteins extracted from the *Nicotiana benthamiana* overexpressing *P19* with *XCP1-His* or *P19* with *XCP1^{C161A}-His* genes. Each step during purification was analyzed by immunoblot with anti-His antibody. Coomassie Blue staining shows all purified protein loaded. Lane 1, total protein extracts from *Nicotiana benthamiana* leaves; Lane 2, resuspended protein pellets after acetone precipitation; Lane 3, flow through (FT) after protein loading onto the Ni-NTA column; Lane 4 or 5: fraction washed by wash buffer; Lane 6, eluted proteins. Experiments were repeated for three times with similar results. (**c**) The CNYDase activity with or without adding 400 μM biotin-CNYD-CHO to the Ni-NTA purified proteins from the *Nicotiana benthamiana* overexpressing *P19*, *P19* with *XCP1-His* and *P19* with *XCP1^{C161A}-His* gene. The proteolytic activity assay was performed by incubating 0.5 μg purified protein with 25 μM Ac-CNYD-AMC substrate. RFU of the cleaved fluorophore was measured after 5 h incubation with the substrate. Values are means ± SD of three independent experiments. Each experimental result was obtained by using five *Nicotiana benthamiana* plants overexpressing proteins transiently. *P* values were calculated by one-tailed unpaired *t*-test (***, $P < 0.001$).

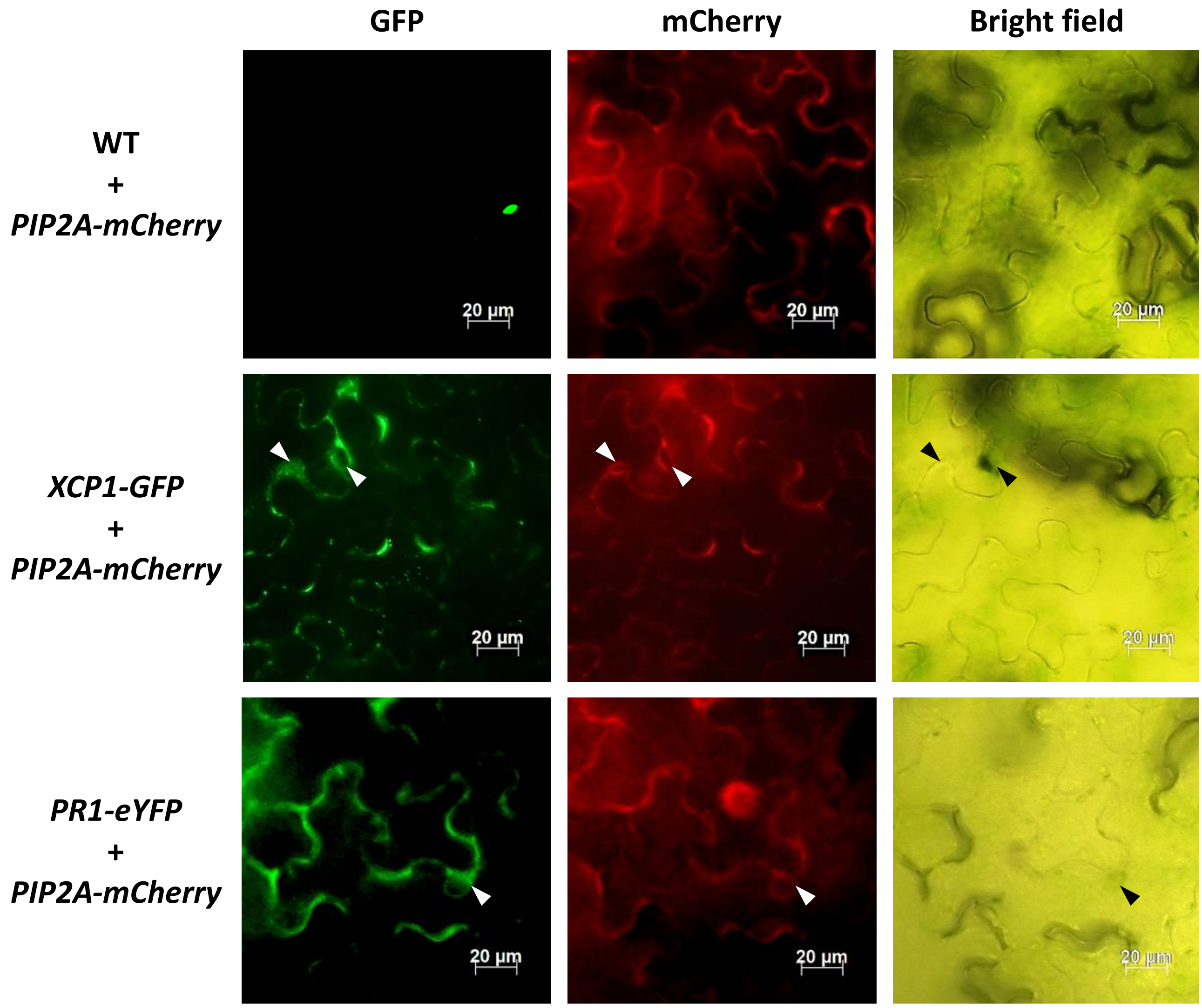

Supplementary Fig. 12. Localization of XCP1-GFP and PR1-eYFP proteins in *Nicotiana benthamiana* leaves subjected to plasmolysis.
The subcellular localization of XCP1 and PR1 were examined by transforming the *2x35S::XCP1-GFP* or *35S::PR1-eYFP* along with *35S::PIP2A-mCherry* in *Nicotiana benthamiana* leaves using *Agrobacterium* infiltration. After 1 M mannitol treatment, the location of overexpressed proteins under plasmolysis in *Nicotiana benthamiana* leaves were detected by fluorescence microscopy. PIP2A-mCherry was used to mark plasma membrane. The arrows indicate the apoplastic regions. Bar = 20 μm. Experiments were repeated for three times with similar results.

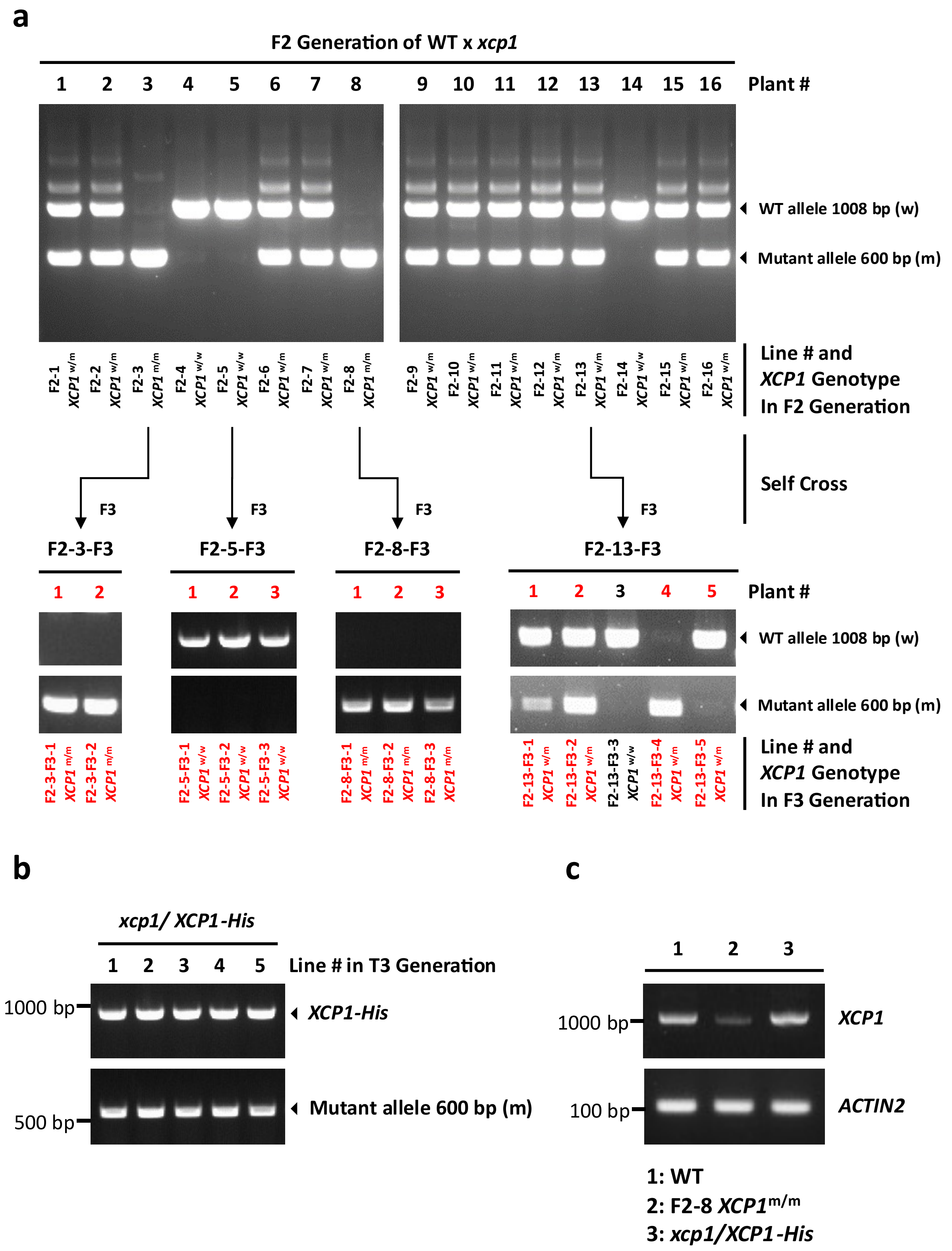

Supplementary Fig. 13. Genotyping and gene expression of the *XCP1* in the crossed or mutated plant lines.

(**a**) Genotyping for the *XCP1* zygosity in F2 and F3 generation of wild-type (WT) and *xcp1* crossed *Arabidopsis* plant (WT × *xcp1*) lines. Size marked for (w) wild-type *XCP1* and (m) mutated *XCP1* allele was 1008 bp (full length of *XCP1*) and 600 bp (T-DNA inserted *XCP1*), respectively. F2 lines of F2-1, F2-2, F2-6, F2-7, F2-9, F2-10, F2-11, F2-12, F2-13, F2-15 and F2-16 were identified as

heterozygous mutated *XCP1* (*XCP1*$^{w/m}$) line. F2-3 and F2-8 were identified as homozygous mutated *XCP1* (*XCP1*$^{m/m}$) line. F2-4, F2-5 and F2-14 were identified as homozygous wild-type *XCP1* (*XCP1*$^{w/w}$) line. F2-3, F2-5, F2-8 and F2-13 were selected to cultivate F3 lines of F2-3-F3, F2-5-F3, F2-8-F3 and F2-13-F3 and genotyped. The selected plant # in each F3 progeny were in red color. The used primers were listed in Supplementary Table 3. Experiment was performed one time. (**b**) Genotyping for the *XCP1* zygosity in T3 generation of *xcp1/XCP1-His*. Experiments were repeated for three times with similar results. (**c**) The expression of the *XCP1* in the wild-type (WT), *XCP1*$^{m/m}$ (F2-8) and *XCP1-His* complemented *xcp1* (*xcp1/XCP1-His*) plants determined by RT-PCR (25 cycles). The expression of *ACTIN2* was used as loading control. Experiments were repeated for two times with similar results.

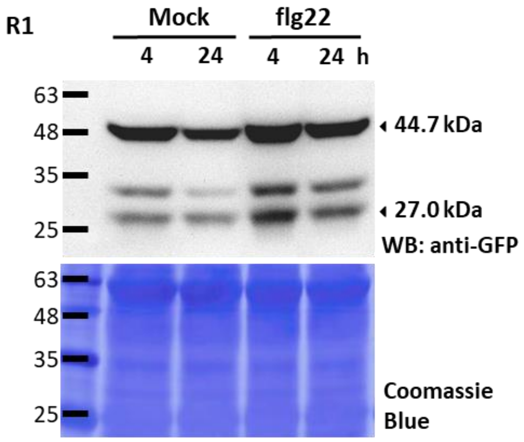

Replicate 1 (R1)	Mock (Control)		flg22	
Time (min)	4	24	4	24
Band intensity of PR1-eYFP	18200	16124	21155	16955
Band intensity of CAPE9-eYFP	6691	5818	10322	7865
%Cleavage [CAPE9-eYFP/ (PR1-eYFP+CAPE9-eYFP)]	26.9	26.5	32.8	31.7
Fold change (Experimental vs. Control)	1.0	1.0	1.2	1.2
Log_2 Fold change (Experimental vs. Control)	0.0	0.0	0.3	0.3

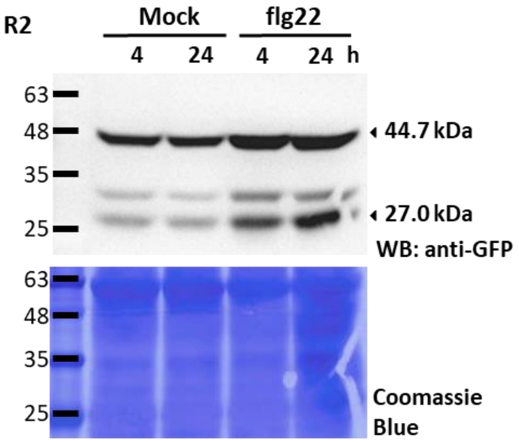

Replicate 2 (R2)	Mock (Control)		flg22	
Time (min)	4	24	4	24
Band intensity of PR1-eYFP	10872	10327	15376	15913
Band intensity of CAPE9-eYFP	3734	3622	8355	10381
%Cleavage [CAPE9-eYFP/ (PR1-eYFP+CAPE9-eYFP)]	25.6	26.0	35.2	39.5
Fold change (Experimental vs. Control)	1.0	1.0	1.4	1.5
Log_2 Fold change (Experimental vs. Control)	0.0	0.0	0.5	0.6

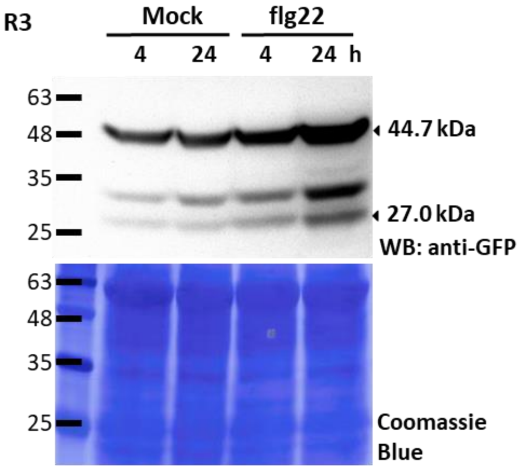

Replicate 3 (R3)	Mock (Control)		flg22	
Time (min)	4	24	4	24
Band intensity of AtPR1-eYFP	12423	17768	23136	26912
Band intensity of AtCAPE9-eYFP	1471	2472	4280	5751
%Cleavage [AtCAPE9-eYFP/ (AtPR1-eYFP+AtCAPE9-eYFP)]	10.6	12.2	15.6	17.6
Fold change (Experimental vs. Control)	1.0	1.0	1.5	1.4
Log_2 Fold change (Experimental vs. Control)	0.0	0.0	0.6	0.5

Supplementary Fig. 14. Immunoblot of the protein extract from *PR1-eYFP* transgenic plant seedlings treated with flg22 using anti-GFP.

The seedlings were immersed in medium with 10 mM $MgSO_4$ (Mock), 500 nM flg22 in 10 mM $MgSO_4$ for 4 and 24 h. The sizes of the intact PR1-eYFP and the putative AtCAPE9 fragment were estimated to be ~44.7 and ~27.0 kDa, respectively. The percentage of the PR1-eYFP cleavage (%Cleavage) was calculated by the band intensity of AtCAPE9-eYFP divided by the sum of the PR1-eYFP and AtCAPE9-eYFP band intensities [(AtCAPE9-eYFP)/(PR1-eYFP+AtCAPE9-eYFP)], detected by immunoblot using anti-GFP. Coomassie Blue staining shows total protein loaded. The band intensities in immunoblots were measured by ImageJ.

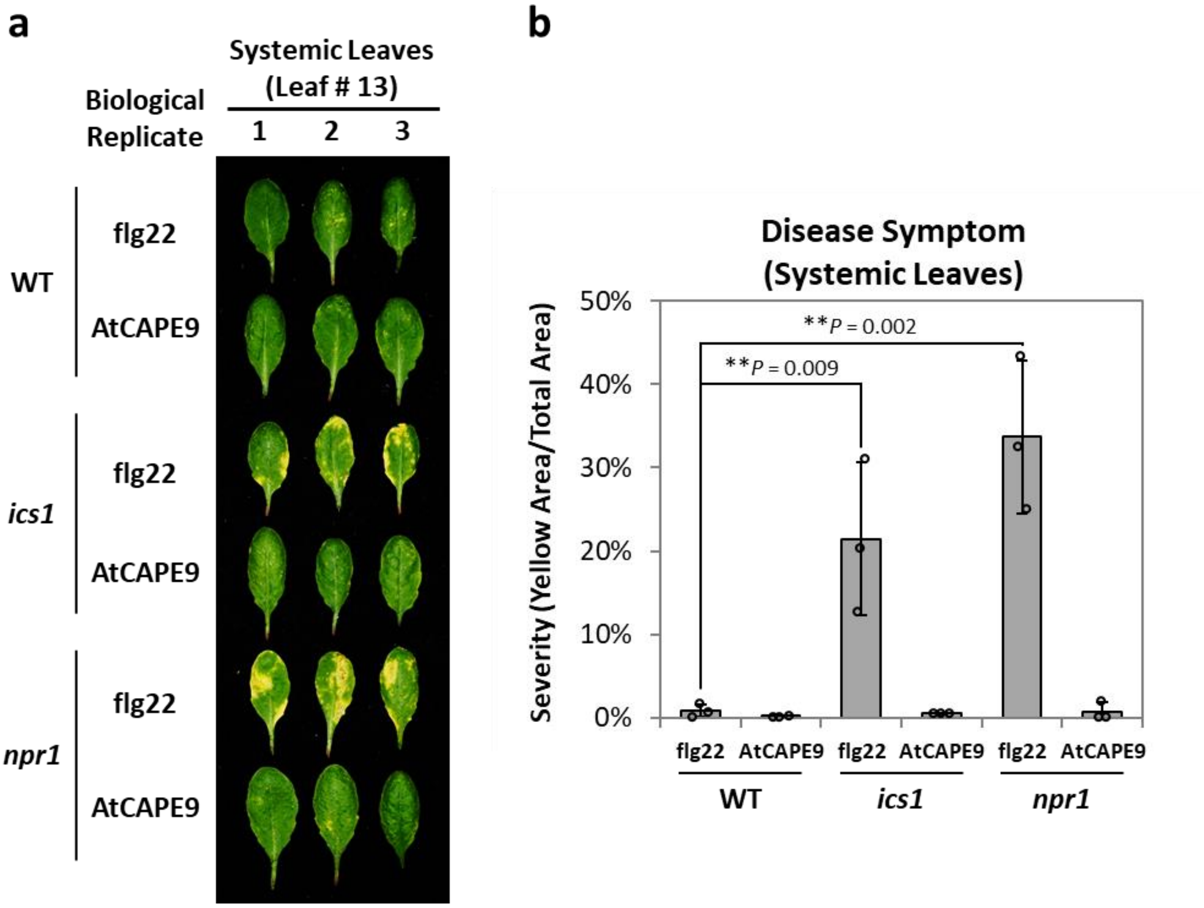

Supplementary Fig. 15. Systemic immunity of wild-type, *ics1* and *npr1* elicited by local treatment of pathogen elicitor flg22 or phytocytokine AtCAPE9.

(**a**) The disease symptom of the *Pst* DC3000-infected systemic leaves of wild-type (WT), *ics1* and *npr1* mutant pre-infiltrated local leaves with flg22 or AtCAPE9. The local pre-infiltrations were performed on the leaf # 7-11 as defined in Fig. 2 and other leaves without infiltration were used as systemic leaves. After 2 days of local infiltration, both local and systemic leaves were inoculated with *Pst* DC3000 for 5 days and collected systemic leaves for disease symptom assessment. (**b**) Levels of disease severity of systemic leaves were measured by the percentage of yellow area in total area for the corresponding photographs using the PIDIQ software. Values are means ± SD of three biological replicates. P values were calculated by one-tailed unpaired t-test (**, $P < 0.01$).
