# Supplementary material for: XCP1 cleaves Pathogenesis-related protein 1 into CAPE9 for systemic immunity in Arabidopsis
Source: Nat Commun. 2023 Aug 4;14:4697. doi: 10.1038/s41467-023-40406-7 (PMC10403534; doi:10.1038/s41467-023-40406-7)
Supplement: Supplementary file 3 — Reporting Summary [file 41467_2023_40406_MOESM3_ESM.pdf]

nature portfolio

Corresponding author(s): Yet-Ran Chen

Last updated by author(s): Jul 20, 2023

Reporting Summary

Nature Portfolio wishes to improve the reproducibility of the work that we publish. This form provides structure for consistency and transparency in reporting. For further information on Nature Portfolio policies, see our Editorial Policies and the Editorial Policy Checklist.

Statistics

For all statistical analyses, confirm that the following items are present in the figure legend, table legend, main text, or Methods section.

n/a	Confirmed	
[ ]	[x]	The exact sample size (*n*) for each experimental group/condition, given as a discrete number and unit of measurement
[ ]	[x]	A statement on whether measurements were taken from distinct samples or whether the same sample was measured repeatedly
[ ]	[x]	The statistical test(s) used AND whether they are one- or two-sided<br>*Only common tests should be described solely by name; describe more complex techniques in the Methods section.*
[ ]	[x]	A description of all covariates tested
[ ]	[x]	A description of any assumptions or corrections, such as tests of normality and adjustment for multiple comparisons
[ ]	[x]	A full description of the statistical parameters including central tendency (e.g. means) or other basic estimates (e.g. regression coefficient) AND variation (e.g. standard deviation) or associated estimates of uncertainty (e.g. confidence intervals)
[ ]	[x]	For null hypothesis testing, the test statistic (e.g. *F*, *t*, *r*) with confidence intervals, effect sizes, degrees of freedom and *P* value noted<br>*Give P values as exact values whenever suitable.*
[x]	[ ]	For Bayesian analysis, information on the choice of priors and Markov chain Monte Carlo settings
[x]	[ ]	For hierarchical and complex designs, identification of the appropriate level for tests and full reporting of outcomes
[ ]	[x]	Estimates of effect sizes (e.g. Cohen's *d*, Pearson's *r*), indicating how they were calculated

Our web collection on statistics for biologists contains articles on many of the points above.

Software and code

Policy information about availability of computer code

Data collection	MS data were all collected using Thermo Xcalibur (V4.1.1.31.9 for QExactive HF; V2.1 for LTQ Orbitrap XL; V2.2 for LTQ Velos Pro and Orbitrap Elite). For the analysis of western blot image, the Chemstudio Plus software Vision Works 9.1 was used. For the DNA/RNA quantification, the NanoDrop 1000 Spectrophotometer software ND-1000 V3.7.1 was used. For the agarose gel imaging, the Bio-Rad GelDoc XR+ with software Quantity One Version 4.6.8 Build 027 was used. For qPCR experiment, 7500 FAST REAL TIME PCR SYSTEM with 7500 software v2.3 was used. For XCP1 enzyme activity and kinetic assay, the BioTek software version 3.08 was used to record the fluorescence signal. For co-localization experiments, Carl Zeiss AxioImagerZ1 software AxioVs40 V 4.8.2.0 was used.
Data analysis	For peptide identification, the MS raw data were converted into Mascot generic format (.mgf) with MSconvertGUI (64-bit). The Mascot MS/MS ion search (Matrix Science, server version 2.3) with the Mascot daemon software (2.6.0) was used for non-enzyme specific search. The peptide or SA quantification was performed by peak area integration using Thermo Xcalibur (V4.1.1.31.9) Qual Browser. The ImageJ version 1.53t was used for protein band quantification to calculate the percentage of AtPR1-eYFP cleavage. The ImageJ version 1.52a with PIDIQ release v0.0.1 plug-in was used for measuring the disease symptom of plant leaves.

For manuscripts utilizing custom algorithms or software that are central to the research but not yet described in published literature, software must be made available to editors and reviewers. We strongly encourage code deposition in a community repository (e.g. GitHub). See the Nature Portfolio guidelines for submitting code & software for further information.

Data

Policy information about availability of data

All manuscripts must include a data availability statement. This statement should provide the following information, where applicable:

- Accession codes, unique identifiers, or web links for publicly available datasets
- A description of any restrictions on data availability
- For clinical datasets or third party data, please ensure that the statement adheres to our policy

The data that support the findings of this study are available within this manuscript and its Supplementary Information and Supplementary Data file. MS raw data for peptidomic analysis were deposited to the ProteomeXchange Consortium via the PRIDE partner repository with the dataset identifier PXD041898 [https://proteomecentral.proteomexchange.org/cgi/GetDataset?ID=PXD041898]. MS raw data for targeted MS analysis using PRM method were deposited to the MassIVE with dataset identifier MSV000091833 [ftp://massive.ucsd.edu/MSV000091833/]. All materials including plant lines, bacteria strains, plasmids and primers used in this study were described in the Supplementary Information. All the unprocessed data, gels and blots were provided in the Supplementary/Source Data file. Data or material used in this paper is also available from the corresponding author upon request.

Human research participants

Policy information about studies involving human research participants and Sex and Gender in Research.

Reporting on sex and gender	*Use the terms sex (biological attribute) and gender (shaped by social and cultural circumstances) carefully in order to avoid confusing both terms. Indicate if findings apply to only one sex or gender; describe whether sex and gender were considered in study design whether sex and/or gender was determined based on self-reporting or assigned and methods used. Provide in the source data disaggregated sex and gender data where this information has been collected, and consent has been obtained for sharing of individual-level data; provide overall numbers in this Reporting Summary. Please state if this information has not been collected. Report sex- and gender-based analyses where performed, justify reasons for lack of sex- and gender-based analysis.*
Population characteristics	*Describe the covariate-relevant population characteristics of the human research participants (e.g. age, genotypic information, past and current diagnosis and treatment categories). If you filled out the behavioural & social sciences study design questions and have nothing to add here, write "See above."*
Recruitment	*Describe how participants were recruited. Outline any potential self-selection bias or other biases that may be present and how these are likely to impact results.*
Ethics oversight	*Identify the organization(s) that approved the study protocol.*

Note that full information on the approval of the study protocol must also be provided in the manuscript.

Field-specific reporting

Please select the one below that is the best fit for your research. If you are not sure, read the appropriate sections before making your selection.

[x] Life sciences [ ] Behavioural & social sciences [ ] Ecological, evolutionary & environmental sciences

For a reference copy of the document with all sections, see nature.com/documents/nr-reporting-summary-flat.pdf

Life sciences study design

All studies must disclose on these points even when the disclosure is negative.

Sample size	No calculation for sample size was performed. Sample sizes were chosen based on our experiences on similar experiments, published work (Chen et. al., Plant Cell 2014, Chien et. al., J. Exp Bot. 2015) and availability of alternative evidences in this work. Each result was concluded by at least three individual samples using the same or alternative experiments to result in scientific significance.
Data exclusions	No data were excluded in the analyses.
Replication	All data can be replicated. The number of replicates performed are indicated in the figure legends or method.
Randomization	All the samples used for treatment comparison in this study were grown in the same condition and randomly chosen for different treatments. For the SAR experiment, the local-infiltrated leaves were not randomly chosen. We fixed the leaf # 7-11 for the local treatment and remaining leaves were used as systemic leaves for the phenotype observation to minimize the age variation of the plant leaves.
Blinding	No formal blinding was used throughout experiments. Only the instrument operators were blinded to group allocation during data collection for instrumental analysis.

Reporting for specific materials, systems and methods

We require information from authors about some types of materials, experimental systems and methods used in many studies. Here, indicate whether each material, system or method listed is relevant to your study. If you are not sure if a list item applies to your research, read the appropriate section before selecting a response.

Materials & experimental systems

n/a	Involved in the study	
☐	☒	Antibodies
☒	☐	Eukaryotic cell lines
☒	☐	Palaeontology and archaeology
☒	☐	Animals and other organisms
☒	☐	Clinical data
☒	☐	Dual use research of concern

Methods

n/a	Involved in the study	
☒	☐	ChIP-seq
☒	☐	Flow cytometry
☒	☐	MRI-based neuroimaging

Antibodies

Antibodies used	anti-His antibody (mouse, 1:5000; #PPT-66005-1, Biotools), anti-GFP antibody (mouse, 1:5000; #11814460001, Roche), anti-AtPR1 antibody (Rabbit, 1:2500, #AS10687, Agrisera)
Validation	The relevant validations of the antibodies we used are provided by the manufacturer's websites.
